# Influence of Passive Stiffness of Hamstrings on Postural Stability

**DOI:** 10.1515/hukin-2015-0006

**Published:** 2015-04-07

**Authors:** Michał Kuszewski, Rafał Gnat, Grzegorz Sobota, Andrzej Myśliwiec

**Affiliations:** 1 Department of Kinesitherapy and Special Methods of Physiotherapy, Academy of Physical Education, Katowice, Poland.; 2 Department of Human Motor Behavior, Academy of Physical Education, Katowice, Poland.

**Keywords:** stability, muscle stiffness, postural control, margin of safety

## Abstract

The aim of the study was to explore whether passive stiffness of the hamstrings influences the strategy of maintaining postural stability. A sample of 50 subjects was selected; the final analyses were based on data of 41 individuals (33 men, 8 women) aged 21 to 29 (mean = 23.3, SD = 1.1) years. A quasi- experimental ex post facto design with repeated measures was used. Categories of independent variables were obtained directly prior to the measurement of the dependent variables. In stage one of the study, passive knee extension was measured in the supine position to assess hamstring stiffness. In stage two, the magnitude of postural sway in antero-posterior direction was measured, while varying the body position on a stabilometric platform, both with and without visual control. The margin of safety was used as a measure of postural control. The magnitude of the margin of safety increased significantly between the open-eye and closed-eye trials. However, although we registered a visible tendency for a larger increase of the margin of safety associated with lower levels of passive hamstrings stiffness, no significant differences were found. Therefore, this study demonstrated that hamstring stiffness did not influence the strategy used to maintain postural stability.

## Introduction

Postural stability is defined as the ability to maintain an erect posture of the body. A similar term, which also reveals a dynamic aspect of postural stability, is balance; this describes the dynamics of body posture to prevent falling. Both postural stability and balance are related to the inertial forces acting on the body and the inertial characteristics of body segments (Winter, 1995). Both are also dependent on central nervous system (CNS) activity, which should provide fast and adequate responses to alternating postural requirements.

The major role in postural control is ascribed to proprioceptive, visual and vestibular input (Fitzpatrick and McCloskey, 1994). These receptors provide a continuous flow of information to the CNS which forms the basis for postural adjustments. Modifications performed on the proprioceptive, visual and vestibular input, and their influence on postural stability, are well documented (Massion, 1992; Maurer et al., 2000).

All actions initiated by the CNS have their mechanical effect using active properties of muscle tissue. This is associated with the excitability of the motoneuron pool and becomes visible as a modulation of muscle stiffness (Feldman, 1966; Latash, 1993). Reports on the influence of alternating active muscle stiffness on postural stability are scarce (Kuczyński, 2001).

Besides this, it seems that passive properties of muscle tissue may also contribute to the process of maintaining postural stability; however, these properties are almost completely neglected by researchers. It must be stressed that during passive stretch, skeletal muscles exhibit measurable resistance even when their motoneurons are quiescent and their myofibers are not actively contracting (Schleip et al., 2006). Moreover, such passive muscle stiffness provides the necessary stabilizing force at once, before the shortest reflex response can be initiated. Therefore, it must always be considered by the CNS in all processes associated with maintaining balance (Winter et al., 1998). This argument indicates that different levels of passive muscle stiffness may be related to different postural strategies activated by the CNS.

Using the model of reverse pendulum, various authors have indicated the important role of the ankle joint muscles in postural control (Fitzpatrick et al., 1992; Winter et al., 2001). Modulation of the ankle joint muscle stiffness allows to maintain balance during anterior and posterior excursions of the center of mass (Winter et al., 2001). Others also claim that control of the lumbar spine and the whole lumbo-pelvo-hip region is equally important in relation to stability and balance (Moorhouse and Granata, 2007). This is the main rationale, in the present study, for choosing the hamstrings as the target muscle group. It is well known that these muscles contribute to pelvic and lumbar control in the sagittal plane (Vleming et al., 1997). In addition, the percentage of people showing increased passive stiffness of the hamstrings is high (Gajdosik et al., 1990) and the measurement technique is relatively easy (Gnat et al., 2010).

To understand our hypothesis, it is necessary to mention three levels of stability control as described by Richardson and colleagues (Richardson et al., 2004); these are: the local or segmental level (e.g. stabilization of the single joint), the level of spinal orientation (e.g. overall orientation of the pelvis or lumbar spine), and the global level (e.g. whole-body equilibrium). In earlier studies we demonstrated how passive stiffness of the hamstrings changes together with improved local stability (Kuszewski et al., 2009); in other words, we have shown how the local level of stability control determines the spinal orientation level (stiffness of the hamstrings is assumed to play a role in controlling lumbar and pelvic orientation).

The aim of the current study was to explore the hypothesis that passive stiffness of the hamstrings (the spinal orientation level) influences the strategy of maintaining postural stability (the global level).

## Material and Methods

### Subjects

A convenience sample of young individuals was tested against inclusion and exclusion criteria. In the first stage a purposive sampling strategy was applied. The established inclusion criteria were: age between 18 and 30 years, absence of any pain or injury (requiring medical advice, bed rest or hospitalization) within the musculoskeletal system during the study period and in the period one month prior to the experiment, no previous history of serious injuries and dysfunctions (e.g. requiring hospitalization or surgical treatment), and no medication which might have influenced the ability to maintain balance. Exclusion criteria were: pain or inability to relax muscles during passive knee extension in the supine position (i.e. the PKES test described below), and inability to maintain balance on the stabilometric platform.

A member of the research team invited individuals to participate in the research and provided them with appropriate verbal and written information about the study. The subjects had an opportunity to ask questions about the objectives and procedures of the study. Verbal and written consent was obtained before the group assignment began (in accordance with the Declaration of Helsinki); this also met the criteria for informed consent as outlined by the institutional Biomedical Research Ethics Committee. After an introductory interview, a sample of 50 subjects was selected. During the research procedure there were 9 dropouts. Therefore, the final analyses were based on the data of 41 individuals (33 men, 8 women) aged 21 to 29 years (mean 23.3 ± 1.1) with body height of 177.2 ± 7.2 cm, and body mass of 73.5 ± 9.2 kg.

### Design

A quasi-experimental ex post facto design with repeated measures was used. Categories of independent variables were obtained directly prior to measurement of the dependent variables. Measurement of the PKES test and anthropometric measurements were performed during stage one of the research. During stage two, the magnitude of postural sway in the antero-posterior direction was measured in varying positions of the body. These two stages took place in two different rooms; during testing the researchers had no contact with each other and were not aware of the results obtained by their colleagues.

### Instrumentation

Measurement of the PKES test was performed using a digital inclinometer (Saunders Group Inc., Chaska, USA) and a handheld dynamometer (MicroFET2, Hoggan Health Industries, Draper, USA) (see below).

**Figure 1:**
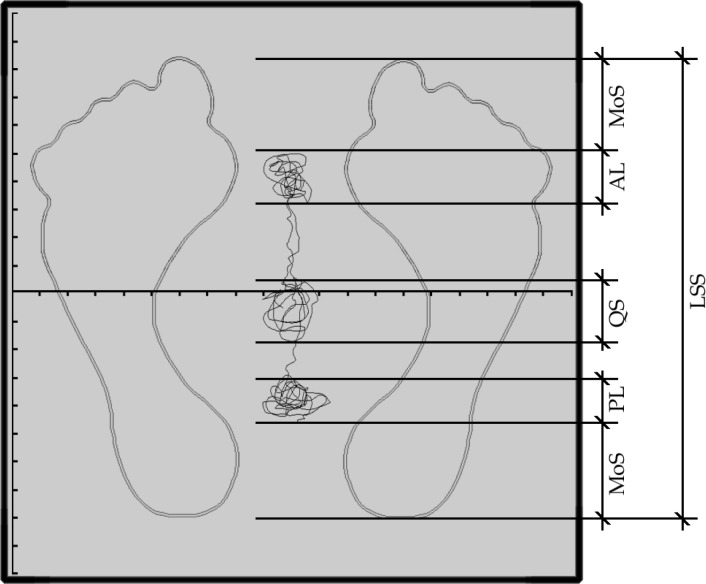
Schematic presentation of the stabilometric platform. Excursions of the center of pressure during quiet standing (QS), anterior lean of the body (AL) and posterior lean of the body (PL) in antero-posterior direction are shown, as well as the anterior and posterior margins of stability (MoS) and overall length of the support surface (LSS)

Stage two included two similar trials performed on a computer-controlled static stabilometric platform (Kistler 9865C; Kistler Instrumentate AG, Winterthur, Switzerland) at an acquisition frequency of 100 Hz. A two-dimensional coordinate system was plotted on the surface of the platform with its center placed exactly in the central point of the platform (Figure 1). This allowed us to precisely reproduce the position of the feet in the second trial performed on the same platform, as well as to measure the length and width of the support surface.

### Experimental procedures

#### Stage one

Stiffness of the hamstrings was evaluated using the PKES as described by Davis et al. (2005) and Gnat et al. (2010). Briefly, this test was started in the supine position with the hip joint and knee on the tested side flexed to 90°. A special custom-made support was placed on the couch to maintain exactly 90° flexion of the hip joint. From this position a passive extension of the knee was performed. The subject’s task was to signal a feeling of ‘strong, but tolerable stretch’ in the area of attachments or muscle bellies of the hamstrings. The angle between the anterior surface of the shin and the horizontal was registered. During the test, the non-tested lower extremity and the pelvis were stabilized by means of firm manual pressure applied by the assistant in the area of the anterior superior iliac spine and middle-anterior aspect of the thigh on the non-tested side.

For the PKES test the angle was measured using a digital inclinometer (Saunders Group); the tested accuracy of the device proved to be ±1°. The inclinometer was placed on the flat anterior surface of the tibia in the middle of the distance between the apex of the patella and the ankle joint at the height of the malleoli measured lying supine. Since stiffness is the ratio of change in muscle length to the value of the force causing this change, and we used the PKES as the measure of change in length, some other measure to control the force was needed. We registered the value of force used for passive extension of the knee in a moment when the subjects signalled a feeling of ‘strong, but tolerable stretch’. This value was consequently used during re-tests. To achieve this, the handheld dynamometer (MicroFET2) was used. When extending the knee, it was placed between the researcher’s hand and the posterior aspect of the shin (on a relatively flat surface below the bellies of the gastrocnemius and above the Achilles tendon) of the subject. This measurement was performed three times to test its reliability. Subsequent statistical analyses were based on the mean value of these three repetitions.

The PKES has proven to be a valid and reliable measure of hamstring stiffness; the intratester reliability of the PKES is reported to be 0.99 using the inclinometer method (Sullivan et al., 1992), and 0.98 using a universal goniometer (Webright et al., 1997; Ford et al., 2005).

#### Stage two

Stage two of the research was performed directly after the PKES test. To minimize any disturbing sensory input, the second test took place in a separate room isolated from external noise and with only one researcher and one subject inside the room. Subjects wore socks so that the cold metal surface of the stabilometric platform did not influence muscle stiffness via activation of the gamma system. The subjects performed two trials on the platform during which data on postural sway in antero-posterior direction were recorded. After stepping onto the platform the subject assumed a comfortable position with their feet; this position was registered using the platform’s coordinate system. The length of the support surface (LSS, Figure 1) was also measured with accuracy of 1 mm.

The first trial was the open-eye (OE) trial which was followed by the closed-eye (CE) trial. After initial 5 s of quiet standing the subject’s task was to maximally lean their bodies in anterior direction without flexing either hip joints or lumbar spine, and keeping their heels in constant contact with the ground. At the 30^th^ s of the trial they resumed quiet standing and at the 35^th^ s they maximally leaned their body in the posterior direction keeping their toes in constant contact with the ground. At the 60^th^ s the OE trial ceased. When the subjects bent their trunk or hips during the measurement, the procedure was repeated and the remark was addressed to them to remain erect.

The set-up for the CE trial was exactly the same, however, there was no visual input (i.e. the eyes were closed). Sixty seconds of relaxation were allowed between the two trials. For the second trial, the initial position of the feet was reproduced precisely.

Before starting the OE trial the whole sequence was thoroughly explained to the subjects. During the performance clear verbal commands were provided by the researcher. When losing proper contact with the ground of either the heels or the toes, the particular trial was repeated.

### Analysis

According to the outcomes of the PKES test (mean value of the right and left sides) subjects were divided into three groups reflecting the different levels of hamstring passive stiffness (categories of independent variables):

- PKES > 85^º^; group 1: normal passive stiffness (8 subjects, mean = 87.54^º^, SD = 1.48^º^)
- 85^º^ > PKES > 75^º^; group 2 moderate passive stiffness (14 subjects, mean = 81.71^º^, SD = 2.88^º^)
- 75^º^ < PKES; group 3: high passive stiffness (19 subjects, mean = 65.82^º^, SD = 4.69^º^)

**Figure 2:**
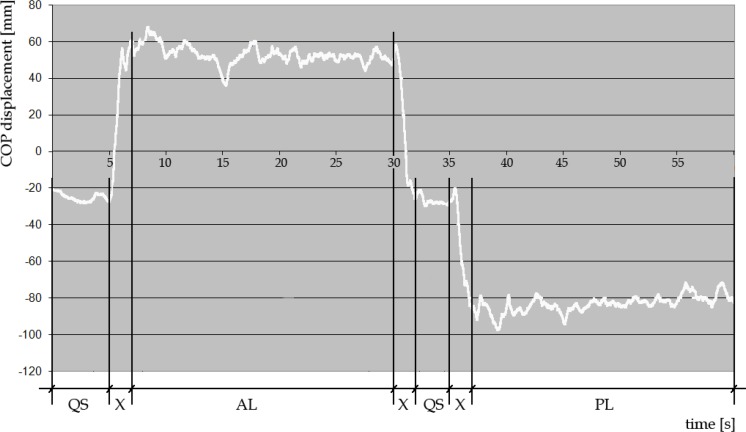
An individual, representative record of excursion of the center of pressure obtained during a single trial on the stabilometric platform. Mean values from sections QS (quiet standing; 5 and 3s) AL (anterior lean of the body; 21s) and PL (posterior lean of the body; 23s) were used in further analysis. The two-second X sections were treated as transitions and were excluded from analysis

It is commonly assumed that the amplitude of postural sway is a valid measure of postural stability. Błaszczyk et al. (1994) proposed a division of the postural sway area into several zones governed by different balance strategies. In their system, proper postural control is reflected not only in a small postural sway area in quiet standing but also in the ability to approximate the center of pressure (COP) either to the anterior or to the posterior border of the support surface. According to these authors the distance separating the COP from the border is called the margin of safety (MoS) (Figure 1) (Błaszczyk et al., 1994). In our sample we used the MoS as a measure of postural control; for this we used the following values:

- length of the support surface (LSS) as measured from the platform coordinate system (Figure 1)
- mean amplitude of postural sway in quiet standing (QS) as calculated from the stabilometric platform raw data (section QS in Figure 2)
- mean amplitude of postural sway in anterior leaning of the body (AL) as calculated from the stabilometric platform raw data (section AL in Figure 2)
- mean amplitude of postural sway in posterior leaning of the body (PL) as calculated from the stabilometric platform raw data (section PL in Figure 2)

Then, the following calculations were made:

- antero-posterior range of postural sway (APS = AL + PL)
- range of the margin of safety (MoS = LSS − APS)
- magnitude of the margin of safety as a percentage of the support surface length (MoS% = MoS × 100% ÷ LSS (Figure 1).

In the process of data reduction we used Bioware 2.20 (provided with the Kistler platform) and MS Office Excel 2007. Statistical analyses were performed with Statistica 6.0. First, we calculated the intra-class correlation coefficient (ICC(3,1)) for the outcomes of the PKES test. Then, a mixed model of ANOVA with hamstring passive stiffness as the independent factor (groups 1, 2 and 3) and the types of trial as the repeated factor (open-eye vs. closed-eye) was applied. Finally, we used Pearson’s product-moment correlation. The alpha level was set at 5%.

## Results

Similar to others, our reliability testing for the PKES test also showed an excellent ICC(3,1) with coefficients higher than 0.90 for both lower extremities.

During the OE trial, a similar level of MoS% was registered in the three groups; the magnitude was about 54–55%. After elimination of visual input, the MoS% increased in all three groups with a visible tendency for a larger increase associated with lower levels of passive stiffness of the hamstrings (Table 1).

Analysis of variance showed neither significant main effect for the groups nor significant interaction effect for MoS%. The main effect for the repeated factor was significant F(1,38) = 17.8; p<0.001. During the two trials, no significant correlation was found between the outcomes on the PKES and MoS%.

## Discussion

Under normal circumstances maintaining postural stability is a relatively effortless task. Continuous afferent input from proprioceptive, visual and vestibular sensors is sufficient for the CNS to introduce adequate adjustments. Even restrictions placed upon visual information do not drastically disturb postural stability, although they usually cause larger postural sway as observed during stabilometric assessment (Lord and Menz, 2000; Hytonen et al., 1993).

Our results show that the range of consciously controlled antero-posterior COP excursions became significantly smaller when visual information was eliminated (larger MoS% in Table 1). This finding is, however, not surprising.

Our main aim was to investigate whether the difference in MoS% between the OE and CE trials was of the same magnitude in all our subjects; for this we used passive stiffness of the hamstrings as the discriminating factor. Indeed, we observed larger differences in the MoS% magnitude between the OE and CE trials in individuals with normal passive stiffness of the hamstrings. Together with increasing passive stiffness there was a tendency for this difference to become smaller, which might indicate a change in the strategy used to maintain postural stability. However, because we were unable to demonstrate a significant difference for the interaction effect (ANOVA), no generalizations can be made concerning larger populations.

In this situation we may only cautiously speculate that muscles with a lower level of passive stiffness are not capable of providing adequate proprioceptive input with only slight changes in their length, which occur when either anterior or posterior leaning of the body is performed. This creates a problem for the CNS which cannot be compensated when restrictions are placed on the visual input.

This situation appears to be opposite in subjects showing increased stiffness of the hamstrings. With smaller muscle length change, the CNS is quickly provided with information concerning the joint position. The afferent input from the stiffer muscle seems to be sufficient for the CNS (at least during easy postural tasks), so that there is no need for supplementary visual support.

Increased stiffness of the hamstrings is commonly seen as an unfavourable feature, which may increase the probability of tissue overload and pain (McCarthy and Vicenzino, 2003); moreover, there is evidence that it is likely to be associated with insufficient core stabilization (Kuszewski et al., 2009). The results of the present study can only suggest that muscles with a higher level of stiffness may also play a role in the process of maintaining postural stability.

At this point we would like to refer again to the three levels of stability control (Richardson et al., 2004) mentioned above; these levels may well be connected by certain compensatory relationships. We recently demonstrated that stability training of the deep muscular corset of the pelvis and lumbar spine (which is normally responsible for local stabilization (Richardson et al., 2004)), may reduce passive stiffness of the hamstrings (Kuszewski et al., 2009). It has been reported previously that the inactive deep stabilizing muscular system of the lumbo-pelvic region causes an increase in the level of co-activation of superficial muscles of the trunk aiming to increase trunk stiffness (Richardson et al., 2004). Therefore, it is possible that increased stiffness of the superficial muscles compensates for insufficient performance of the deep system, and that a compensatory link exists between the local level of stability control and the spinal orientation level of stability control.

In the current study we aimed to investigate whether a similar link exists between the spinal orientation level and the global level. We considered a chain of interrelations with deep muscular corset insufficiency at the beginning (i.e. local level of stability control), compensated by increased active (co-activation of antagonistic superficial muscle groups, probably occurring earlier) and passive stiffness of the superficial muscles (i.e. spinal orientation level of stability control), which in turn influence the strategy of maintaining postural stability (i.e. global level of stability control). However, no matter how interesting this hypothesis may be, the current study was unable to provide significant evidence to support it. Nevertheless, it seems worthwhile to continue exploring this topic perhaps using some other aspects of postural stability, or reconsidering the procedure for measuring hamstring stiffness, and/or using more numerous muscle groups in order to establish the global level of stiffness presented by a given individual.

## Conclusions and implications for injury prevention

Considering the results of the current study it is impossible to claim definitely that hamstring stiffness influences the strategy that is used to maintain postural stability. Literature and our previous studies indicate, however, that such interrelations are not unlikely. From the perspective of competitive sports and injury prevention a hypothesis on deregulation of delicate balance between the three levels of stability control and consecutive compensatory changes seems especially interesting. Keeping it in mind coaches, physiotherapists and athletes may find better solutions for injuries treatment and prophylaxis. Sometimes what is most evident (e.g. increased hamstrings stiffness) may be an outcome of (e.g. deep muscular corset insufficiency) or a reason for (e.g. diminished global postural stability) more subtle and potentially more dangerous situations. Further research and evidence is needed to support this conception.

## Figures and Tables

**Table 1:** Magnitude of the margin of safety (as percentage of support surface length) in the open-eye and closed-eye trials on the stabilometric platform and mean difference in the margin of safety between the trials

Group	Open-eye trial (1)	Closed-eye trial (2)	Δ (2) − (1)
1	53.9 ± 8.6	57.4 ± 8.2	3.5 ± 4.3
2	55.7 ± 8.4	58.5 ± 9.5	2.8. ± 3.3
3	54.3 ± 7.7	55.8 ± 7.7	1.5 ± 3.7
